# Hybrid motility mechanism of sperm at viscoelastic-solid interface

**DOI:** 10.21203/rs.3.rs-4284452/v1

**Published:** 2024-04-25

**Authors:** Shobitha Unnikrishnan, Robert Scott, Emmanuel Ogundele, Mohammad Azad, Kenta Ishimoto, Susan Suarez, Chih Kuan Tung

**Affiliations:** North Carolina Agricultural and Technical State University; North Carolina Agricultural and Technical State University; North Carolina Agricultural and Technical State University; North Carolina Agricultural and Technical State University; Kyoto University; Cornell University; North Carolina Agricultural and Technical State University

## Abstract

To fertilize eggs, sperm must pass through narrow, complex channels filled with viscoelastic fluids in the female reproductive tract. While it is known that the topography of the surfaces plays a role in guiding sperm movement, sperm have been thought of as swimmers, i.e., their motility comes solely from sperm interaction with the surrounding fluid, and therefore, the surfaces have no direct role in the motility mechanism itself. Here, we examined the role of solid surfaces in the movement of sperm in a highly viscoelastic medium. By visualizing the flagellum interaction with surfaces in a microfluidic device, we found that the flagellum stays close to the surface while the kinetic friction between the flagellum and the surface is in the direction of sperm movement, providing thrust. Additionally, the flow field generated by sperm suggests slippage between the viscoelastic fluid and the solid surface, deviating from the no-slip boundary typically used in standard fluid dynamics models. These observations point to hybrid motility mechanisms in sperm involving direct flagellum-surface interaction in addition to flagellum pushing the fluid. This finding signifies an evolutionary strategy of mammalian sperm crucial for their efficient migration through narrow, mucus-filled passages of the female reproductive tract.

## Introduction

For mammalian fertilization to succeed, sperm must pass through complicated and often narrow passageways that could influence sperm movement^1,2^. The passages are generally filled by a highly viscoelastic fluid, such as cervical mucus and oviductal fluid^3^. Viscoelastic fluid is both viscous and elastic due to the presence of macromolecules, such as mucins, which create a scaffolding structure that provides elasticity while they are not chemically bonded to each other. As a consequence, the structure eventually deforms (flows). Clinically, however, sperm motility is typically assessed in vitro, commonly in watery media of low viscoelasticity that could alter sperm movement patterns. In this study, we examined the mechanism that thrusts sperm forward in a physical environment that more closely resembles the natural environment to better understand how sperm migrate in vivo; that is, narrow channels filled with highly viscoelastic fluid.

It has long been known that sperm typically swim near liquid-solid interfaces^4^, a tendency primarily attributed to the fact that sperm are “pusher” microswimmers, meaning they propel themselves forward by pushing fluid backward^5–8^. This behavior particularly makes sperm tend to travel along the corners formed by the meeting of two surfaces. In the female tract, microgrooves in the walls can act like corners and thereby provide an effective guidance mechanism for sperm migration^9,10^. There is evidence that microgrooves in the walls of the bovine cervical canal not only guide sperm but also protect sperm from being swept away by cleansing fluid that flows from the uterus through the cervical canal out to the vagina^11,12^. Hence, sperm interaction with surfaces plays a significant role in enhancing their migration through the female tract.

In addition to the effects of surfaces on sperm movement, the mode of sperm locomotion is also highly dependent on the fluid environment through which they swim^13^. When not near solid surfaces, sperm tend to swim using a rolling motion^14^. When sperm arrive near a surface, the motility mode depends on the fluid properties. In a low-viscosity medium, the same rolling motion continues to be observed as sperm swims along a surface. In a high-viscosity or highly viscoelastic fluid, sperm flagella are known to beat two-dimensionally on surfaces^13,15^. Sperm swimming in highly viscoelastic fluids also tend to form dynamic clusters and swim parallel to their close neighbors^14^. To understand how sperm travel in the female tract to reach the fertilization site, it is essential to know how sperm move at the interface of viscoelastic fluid with solid surfaces.

Sperm motility through a more complex mechanism beyond merely pushing surrounding fluids has been proposed on three occasions. In 1972, it was first suggested that the sperm flagellum exhibits movement similar to a snake crawling on a surface, hinting at a motility mechanism akin to that of a snake^16^. (Note that the exact locomotion mechanism of a snake was not clarified until 2009^17^.) More recently, total internal reflection fluorescence microscopy showed that sperm “slithering” using planar-beating flagella were within 1 μm of a solid surface^13^. Although the authors referred to slithering sperm as swimmers, the steric interaction between the sperm and the surface was mentioned as the mechanism that confines the flagella to two-dimensional motion, therefore pointing towards direct flagellum-surface interaction. In one of our recent studies, we found that, in a viscoelastic fluid, sperm engage in stable, long-range collective dynamics in which thousands of sperm move closely together in the same direction^18^, although this “flocking” behavior is theoretically predicted as unstable when the momentum conservation between the microswimmers and the surrounding fluid is taken into account^19^. One possible explanation for the non-conservation of total momentum between the sperm and the fluid is the direct transfer of momentum between the sperm and the surface, further supporting the analogy of slithering/crawling behavior. All of these findings suggest a strong possibility of a direct momentum transfer between the flagellum and the solid surface. The role this momentum transfer plays in motility, however, remains to be explored.

Here, using a microfluidic in vitro model, we examined sperm flagellum interaction with a solid surface. We found evidence that kinetic friction between the sperm flagellum and the surface plays a role in driving the sperm forward. We found it likely that, in a groove-like structure, the sperm flagellum generates thrust through friction from more than one surface simultaneously. At the same time, the part of the flagellum that deviates from the surface pushes fluid backward. By using tracing beads, we obtained a flow field generated by the sperm that bears resemblance to a flow field produced by an idealized pusher swimmer^20,21^, more so when including beads directly pushed by the sperm head or tail. This observation led us to conclude that the polymer solution we used has significant slippage at solid surfaces, challenging the conventional ‘no-slip’ boundary condition typically used in related fluid simulations^21,22^. It highlights the potential for refining existing models to represent sperm movement more accurately in a complex environment.

Overall, our study contributes to a more comprehensive understanding of how sperm can efficiently move within the spatially confined, viscoelastic fluid environments of the female tract. This understanding could lead to improved fertility assessments, novel treatment strategies, and the development of new sperm selection methods.

## Results

### Flagellar dynamics at the interface reveal that kinetic friction from a solid surface pushes sperm forward.

**Figure 1:**
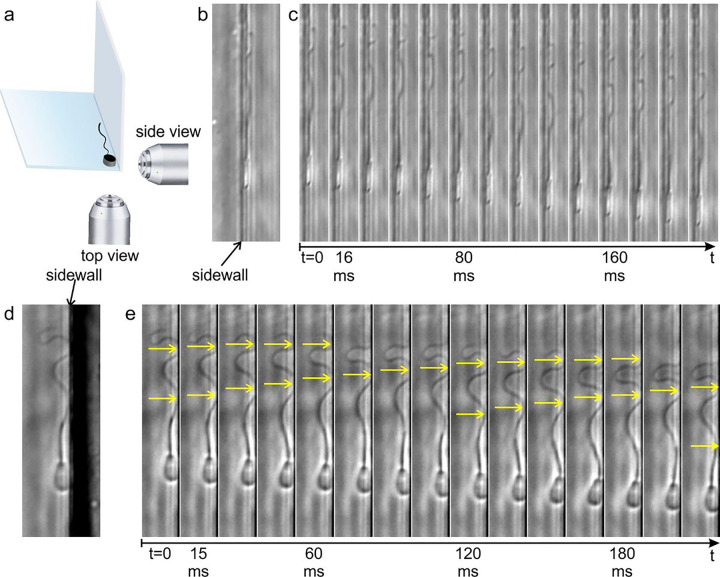
Images of flagellum interaction with a solid substrate show strong evidence of thrust generated from flagellum-surface solid contact. **a** Illustration of sperm moving along a corner, enabling imaging of sperm interacting with different surfaces. Note that only a single objective was used for imaging. The differing perspectives result from the sperm’s natural movements on different surfaces of the corner, allowing us to capture their interactions with the surface from both top and side perspectives without multiple objectives. **b** Side view image of forward progressing sperm. Note that the side wall in the image corresponds to the bottom wall of the illustration. The head appears bright due to phase contrast microscopy. A significant portion of the flagellum can be seen in contact with the surface. **c** A montage of a time-lapse image sequence from the side view. The flagellum contact with the surface is clearly seen moving backward (arrowheads) and appears to generate kinetic friction in the forward direction. **d** Top-view image of a forward-moving sperm. **e** A montage of a time-lapse image sequence from the top view showing that the contact between the flagellum and the sidewall also moved backward (arrowheads).

To study the sperm flagellum interaction with a solid surface, we utilized a microfluidic device with a channel that had a clean-cut corner (see Methods) and was filled with a viscoelastic solution of 1% methylcellulose in sperm TALP medium. The channel enabled visualization of sperm interaction with two different surfaces perpendicular to each other (Fig. 1a). The traditional view of sperm moving along a surface is akin to images taken by the objective below in Fig. 1a or imagery seen in Fig. 1d, which will be referred to as the “top view” in the rest of the text; the images represent a view of the broader surface of the paddle-shaped sperm head. The images taken by the objective to the right in Fig. 1a yield imagery of sperm flagellum close to the surface and shown in Fig. 1b, referred to as the “side view”; the images represent a view of the narrow surface of the head. In reality, we had only one objective, and the side-view images were taken when sperm traveled on the surface parallel to the objective optical axis.

From the side view in Fig. 1b, it can be seen that a substantial portion of the flagellum maintained contact with the surface. The montage of the time-lapse images is shown in Fig. 1c. As the head moved forward (downward in these images), the portion of the flagellum that was in contact with the surface was moving backward (upward) (Supplementary Movie 1). It appeared that the flagellum slid backward on the surface, therefore incurring kinetic friction in the forward direction and becoming a source of the thrust.

The top view of sperm movement has been reported and analyzed before^5,9^. Here, we note that, even in the top view, the flagellum had direct solid contact with the sidewall and with the contact point moving backward, appearing to suggest that kinetic friction in the forward direction is incurred through solid contact (Fig. 1e) (Supplementary Movie 2). This movement pattern was seen on both the upper and lower surfaces of the channel.

### Quantification of the flagellum-surface interaction

**Figure 2:**
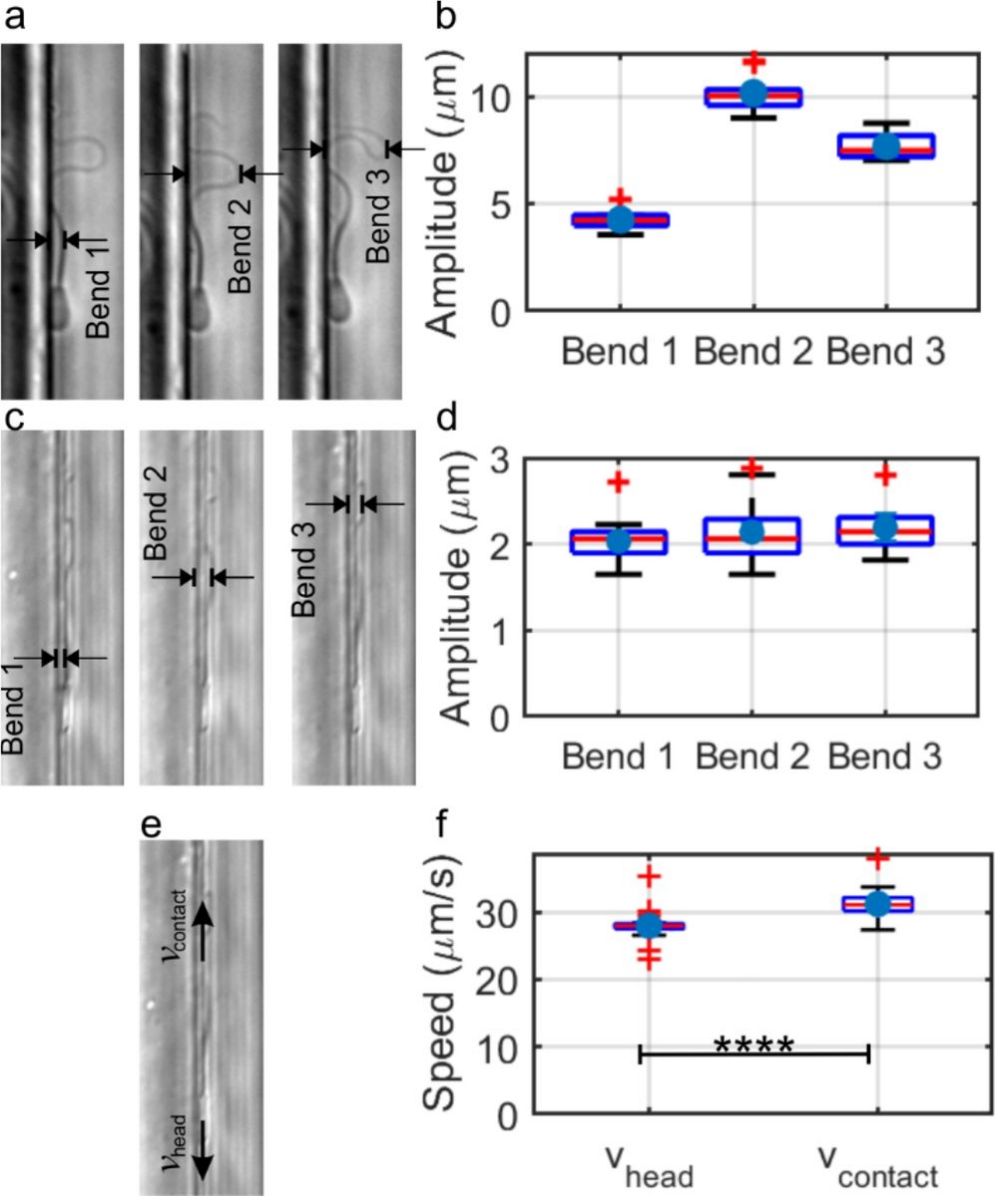
Characterization of the dynamics of flagella. **a** A top view of the sperm highlighting the three distinct bends as it exhibited mostly planar beating. **b** A comparative box plot depicts the amplitude distributions of the three bends formed by flagella in the top view. Error bars represent the standard error of the mean (SEM, n=10). A one-way ANOVA test indicates a significant amplitude difference between all bend types pairs (p < 0.05). **c** A side view of sperm showcases the three distinct bends that the flagellum consistently formed when interacting with the surface. **d** A box plot displays the amplitudes of the three flagellar bends observed in the side view, with an amplitude of around 2 μm. Error bars denote SEM, n=18. ANOVA yields a p-value of 0.195, suggesting no significant difference between them. **e** The side view of sperm movement shows the flagella’s contact point with the surface moving backwards as the sperm head advances. **f** A box plot compares the velocities of the head and flagellum contact. A paired t-test shows a significant difference (p<0.0001), with the flagellum contact consistently moving faster.

To explore the reach and limit of this flagellum-surface motility mechanism, we performed several quantitative measurements of sperm flagellar beating and the flagellum-surface interactions. In Fig. 2a, b, we present our measurements of the flagellar beating amplitudes from the top view. This measurement is important because, if a sperm cell is situated in spatial confinement less than this amplitude, the flagellum can produce kinetic friction from two parallel surfaces, similar to scenarios encountered in confined spaces such as in the female tract, rather than with just one surface.

In the top view, we observed that, along the flagellum, each bend was sequentially generated at the junction with the head and then propagated down its length, resulting in the consistent formation of three distinct propagating mechanical bends along the flagellum (Fig. 2a) (Supplementary Movie 2). Bend 1 is closest to the head and observed around the mid-piece, Bend 2 is around the middle of the principal piece, while Bend 3 is located close to the end of the principal piece or around the end piece. Significant amplitude variations between bends in different stages are evident in the box plot (Fig. 2b), a simple reflection of the beating pattern. Note that the largest amplitude was found to be around 10 μm, suggesting that, in a groove-like structure less than 10 μm wide, the flagellum could generate thrust from friction on both sidewalls.

The side view, on the other hand, reveals three bends with similar amplitudes, as they were found roughly the same distances from the head (Fig. 2c, d) (Supplementary Movie 1), with amplitudes around 2 μm. This value is slightly higher than the 1 μm distance previously reported between the flagellum and the surface^13^, yet not by far. For a narrow slit-like structure with an opening less than 2 μm, such as those found in preovulatory bovine uterotubal junctions ^23^, it appears likely that the flagellum can touch both surfaces.

Finally, from our detailed analysis of the tracking from videos of sperm movement, it was revealed that as the head moved forward, the portion of the flagellum in contact with the surface exhibited backward movement. Further, the speed of the contact point between the flagellum and the surface was higher than the speed of the sperm head (Fig. 2e, f), suggesting that the flagellum slid backward on the surface, further supporting the hypothesis that the kinetic friction between the flagellum and the surface was in the forward direction, thereby providing thrust to the sperm. Moreover, since kinetic friction is solely determined by the force between the two sliding bodies (normal force) and the surface properties (coefficient of friction) and is independent of the relative speed between the sliding objects, this observation seems to imply that the backward traveling of the wave sustained on the flagellum has a function other than generating thrust through friction; otherwise, the energy spent on sustaining the mechanical wave would have been wasted.

### The flow generated in the surrounding fluid by moving sperm does not balance out the forward momentum of the sperm.

**Figure 3:**
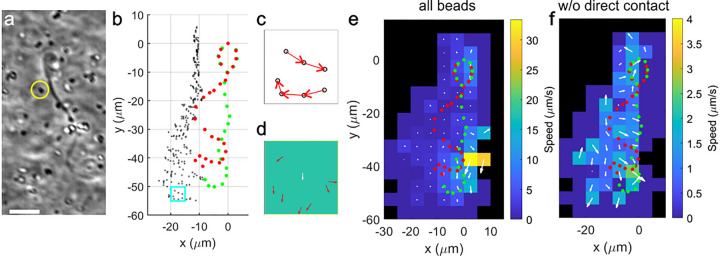
Visualizing fluid dynamics around bovine sperm. **a** Sperm movement in 1% methylcellulose solution with tracer particles. Scale bar: 10 μm. **b** Sperm orientation (head facing upward) and relative tracer positions. The highlighted region shows tracer positions within the same bin. **c** A view of a single bin showing multiple velocity vectors from consecutive tracer locations. **d** A single bin, showing velocity vectors (red arrows) from all tracers averaged into one vector (white arrow) representing the bin. **e,f** These two flow field plots map the flow velocities surrounding a sperm in two dimensions (x and y). The average velocity is depicted by white arrows, indicating flow magnitude and direction. Note that we obtained the flow field plot by averaging velocities over time with no externally applied flow, in addition to spatially averaging velocities within a small bin with respect to the sperm’s relative positions. The overlaid sperm (red and green dots) depicts sperm head and flagellum positions relative to the average flow field, not a moment-by-moment correlation between flagellum and flow field. **e** The average flow field generated by sperm when all tracer particles were included in the calculation, including those directly pushed forward by sperm. **f** The average flow field generated by sperm that excludes tracers pushed directly by the head and the tail.

We previously reported that the flow generated by sperm in highly viscoelastic fluid is less extensive than the flow generated in standard medium^14^. Since the backward propagating wave and the contact point speed both suggest flow generation from the flagellum, to better understand the fluid’s role in sperm motility mechanism, we measured the flow field around moving sperm in 1% methylcellulose solution containing tracer particles. Figure 3 illustrates how the flow field was obtained. We first took raw images of sperm and tracers (Fig. 3a and Supplementary Movie 3). We next tracked the positions of the tracers and the sperm, using these data to determine tracer movement and, consequently, the velocity in real space at different positions relative to the sperm (Fig. 3b). Each tracer movement is represented by a displacement vector and segregated into different bins according to their relative position to the sperm head (Fig. 3c). Velocity vectors from all tracers within the same bin (accumulated throughout the flagellum beating cycles) were then averaged into one velocity vector representing the flow velocity of the bin (Fig. 3d, white arrow), and the results of all bins are shown in Fig. 3e, with additional data shown in Supplementary Fig. 1. In Fig. 3e, the measured flow field roughly resembles an idealized pusher swimmer flow field^24 20,21^, with forward flow around the head, backward flow around the tail, and inward flow on the left hand side, suggesting that the sperm flagellum pushes fluid backward, or “swims,” simultaneously to pushing the solid surfaces. As observed in Supplementary Movie 4, the bead movement in the side view indicates limited flow toward the sperm in the perpendicular direction, which is another resemblance to the ideal pusher swimmer flow field. We estimated the net momentum in the y direction to be (6 ± 9) × 10^−9^ g-μm/s (for details, see Supplementary Analysis). Although the mean value is positive, indicating net forward momentum combined between the sperm and the fluid, the uncertainty is high due to significant cancellation between positive and negative values. The fluid boundary condition may also reduce the negative fluid momentum.

However, we note that the flow field does not look the same when tracers directly pushed forward by the sperm head, and those hit by the flagellum were excluded from the analysis. In this case, the measured flow field became what is shown in Fig. 3f, with generally much reduced flow.

Since the tracer movements were significantly different between those that directly came in contact with the sperm (Supplementary Movie 5) and those without contact (Supplementary Movie 6), we suspect that the no-slip boundary condition was not a good assumption for the interface between the viscoelastic solution and the solid structure of sperm, such as the head and potentially the tail as well.

### The flow profile of the viscoelastic solution reveals a slip boundary at a solid interface.

**Figure 4:**
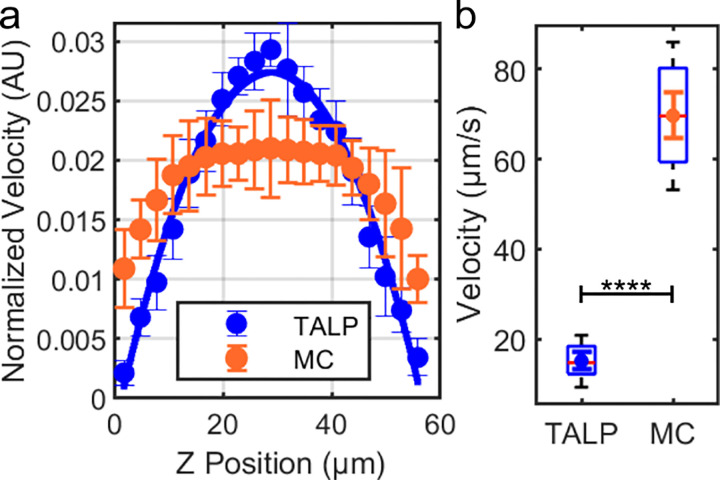
Comparative analysis of fluid flow. **a** Normalized flow velocity profiles for standard TALP medium and 1% methylcellulose (MC) in TALP, revealing a nearly parabolic Poiseuille profile (fitted solid blue line) for TALP and a highly flattened profile for the MC solution. While the MC solution had speed near the solid boundary, roughly half of the peak at the center of the channel, the speed at the wall for TALP alone was found to be an order of magnitude reduced. Error bars: SD. **b** The box plot shows that the flow velocities near the solid boundary are significantly higher for 1% MC solution than for TALP alone. Error bars: SEM, n =10. An independent t-test indicates a significant difference, with p< 0.0001.

As our flow field measurements suggest the existence of a slip boundary of our viscoelastic solution at a solid surface, we decided to explicitly test this possibility. We measured the flow velocity profile of the two sperm media, standard TALP medium and 1% methylcellulose in TALP, under a pressure-driven flow within a rectangular microfluidic channel approximately 60 μm deep and 2.47 mm wide (see Methods). Figure 4 presents the comparative analysis of fluid behavior.

In Fig. 4a, we show the normalized velocity profiles of the standard TALP medium and the 1% methylcellulose solution in TALP. The TALP control profile is very close to the parabolic Poiseuille profile of an ideal Newtonian fluid, while the MC solution profile exhibits significant flattening near the center of the channel, suggesting significant shear-thinning of the fluid. The slight deviation of the TALP control profile from the perfect Poiseuille profile may be attributed to a small shear-thinning property of the bovine serum albumin in TALP^25^.

Regarding the interface boundary conditions, we should focus on the data points close to *z* = 0 or 60 μm. In Fig. 4a, we saw that near a surface, in MC solution, the speed of the tracer particles was found to be 51.89% of the peak speed at the middle of the channel. In TALP, the speed of the tracer particles at the surface was 7.31% of the peak speed observed in the middle of the channel. Figure 4b shows the box plot comparison for the measured speeds near the solid surface, demonstrating a statistically significant higher speed for MC than in TALP control. Note that the imaging depth of our objective was estimated to be 4.375 μm, and therefore, the non-zero mean does not contradict a no-slip boundary. Supplementary Movie 7 shows that some tracer particles did not move with the flow. Overall, the fluid slip at the solid boundary was quite prominent in MC solution (Supplementary Movie 8).

## Discussion

We investigated how bovine sperm move at the viscoelastic fluid-solid interface using a microfluidic model with a 1% methylcellulose solution as the model viscoelastic medium. We present here direct visual evidence of solid-solid interaction between the sperm flagellum and solid surfaces. The observed relative motion between sperm and surface suggests that the kinetic friction experienced by the sperm is in the direction of its forward motion, making it part of the hybrid mechanism, besides swimming, that provides thrust to the sperm.

As sperm traveled along a surface, the flagellum formed a consistent pattern of bends propagating from the flagellar midpiece to the end piece at the tip of the tail. The amplitudes of successive bends demonstrate that when sperm pass through a narrow space (roughly ≤ 2 μm, which is the thickness of the sperm head) filled with highly viscoelastic fluid, the thrust generated through friction likely arises from interactions with surfaces on both sides of the sperm. In the case of the bovine uterotubal junction, sperm may simultaneously contact both sidewalls of microgrooves in the mucosal epithelium^11^. In the case of the oviduct, sperm may pass through the narrow spaces between mucosal folds ^23^. This is particularly intriguing since kinetic friction dissipates energy. The biological rationale for sperm to adopt this motility mechanism that purposefully dissipates energy remains to be seen.

For friction to occur, one of the necessary conditions is a normal force between the two touching surfaces^26^. In the case of a snake slithering, the normal force balances out the weight of the snake from gravity^17^. In other words, snakes cannot slither on a ceiling. Interestingly, the same flagellum-surface interaction was observed on the upper and lower surfaces of the channels in the devices, indicating that the source of the normal force for sperm is NOT from gravity. For all practicality, for a low-Reynolds number swimmer whose inertia is considered negligible^27^, the effects from gravity should not be significant. We suspect that the depletion interaction^28^ is at work here. When two different sizes of objects are randomly distributed in a small molecule solvent (in this case, our medium, molecularly primarily water), the smaller objects (in this case, the polymer macromolecules) have more freedom to move around, and maximize the entropy of the whole suspension, it is probabilistically more likely that the larger objects (in this case the sperm) get “depleted” from the middle of the uniform distribution of the smaller objects. In the current case, depletion from polymers and the subsequent osmotic force provide the interactions needed to form the normal force between the sperm and the surface.

As the head of the sperm advanced along the walls in the channels of the devices, the flagellar contacts with the walls moved backward, interestingly, at a faster rate than the forward motion of the head. While the backward-moving contact points produced kinetic friction with the surface in the direction of the forward movement, this heightened speed of movement seems unnecessary for the generation of the thrust from friction along the wall. This observed phenomenon suggests that the flagellum, particularly the parts deviating from the surface, likely pushes the fluid while, simultaneously, the portion in contact with the surface pushes against the solid surface^29^.

While the flow field pattern we observed could be somehow aligned with that generated by the idealized pusher microswimmer, more resemblance was seen when the tracks of tracers directly pushed forward by the sperm head and tail were incorporated into our analysis. If these beads were excluded from the analysis, we could still see backward moving flow, while the forward flow around the sperm head was reduced. From our momentum analysis, the backward momentum of the fluid was found to be less than the forward momentum of sperm. Further investigation will be needed to verify the split of the thrust from solid-fluid interaction and solid-solid interaction.

Meanwhile, both the flow generated by sperm and our direct measurement of the pressure-driven flow profile indicated that the viscoelastic polymer solution fluid underwent a significant amount of slipping along the surface of the solid. We propose that the slippage is related to the depletion interaction between the polymer chains and the imperfections on the surface^30^, although this phenomenon has not been commonly considered in various microswimmer fluid models^22,31–35^. In short, assuming a no-slip boundary is often a good approximation since solvent molecules scatter randomly when colliding into a solid surface that is microscopically rough. When there are random polymer chains (smaller objects) in the solution, due to entropic effects, polymer chains often do not fill in between all the microscopic solid protrusions (larger objects), therefore forming a thin layer of solvent without polymer, allowing the fluid with polymer chains entangled in it to slip relative easily to the solid surface. The slip boundary we present here is a direct link to the effects of depletion interaction from the polymer, which further strengthens our argument that the depletion interaction leads sperm toward the surface.

Another implication regarding the slip boundary is the interaction between sperm and the fluid. As the viscoelastic polymer solution slips relative to the movement of the sperm, particularly the flagellum, the movement of the flagellum will not push the fluid as efficiently as when pushing a simple saline solution. This is consistent with our earlier report that passing-by sperm generated more fluid movement in the standard medium than in viscoelastic polymeric fluid^14^, and further highlights the advantage for sperm flagellum to engage in near planar beating that facilitates solid-solid interaction.

In a low-viscosity medium, sperm exhibit a rolling motility, whether near or far away from a solid surface^36^. In a high-viscosity or viscoelasticity fluid, the same rolling is seen when sperm are far from a solid surface, yet near planar beating is seen when they are found to be moving along a solid surface. How and why sperm switch between these different motility modes is not well understood^13^. We note here that, in both high-viscosity (Newtonian) and viscoelastic fluids, the fluid rheological properties are achieved by the addition of polymer to the solution; therefore, the effect may well come from the dissolved polymer instead of the viscosity. In fact, if the depletion corresponds to the normal force between the sperm and the solid surface, the same forcing toward the solid surface likely also forces the two-dimensional beating of the flagellum. Furthermore, when sperm engage in this near planar beating motility between the entangled polymer web and the solid substrate, it is possible that sperm follow a thin layer of solvent, allowing them to move with less resistance from the fluid.

In conclusion, we report that, at the interface of a viscoelastic fluid and a solid substrate, sperm propel themselves through a combination of direct flagella-surface contact and conventional swimming (flagella pushing fluid). The solid interaction coincides with the strong tendency of sperm to move near solid boundaries. The natural fluids through which the sperm pass in the female reproductive tract are full of macromolecules (mucins in cervical mucus, for example) and highly viscoelastic^37^. Given the narrow confines of the female reproductive tract, the propulsion from solid-solid interactions may be the predominant force that pushes sperm to reach the fertilization site in mammals.

## Methods

### Media preparation

The standard medium used in this study, Tyrode’s Albumin Lactate Pyruvate (TALP)^38^, was composed of 99 mM NaCl, 3.1 mM KCl, 0.39 mM NaH_2_PO_4_, 25 mM NaHCO_3_, 10 mM HEPES free acid, 2 mM CaCl_2_, 1.1mM MgCl_2_, 25.4 mM sodium lactate, 1 mM/mL sodium pyruvate, 5 mg/mL gentamicin, and 6 mg/mL bovine serum albumin (BSA), titrated with 1 M HCl to a pH of 7.4. Our viscoelastic fluid was made of 1% w/w methyl cellulose (1% MC) in TALP (4,000 cP at 2%) Methylcellulose was added to the medium to add viscoelasticity and closely simulated the conditions of the female reproductive tract, and its weakly elastic nature allows for modeling in numerical simulations^32,39^. The rheological measurements of 1% MC are detailed in Supplementary Fig. 2. 0.35 μm carboxylated polystyrene beads were added to the 1% MC for flow tracing. Carboxylated beads were used because they were found to reduce clumping of plain polystyrene beads in the TALP medium.

### Sperm sample preparation

Bull semen frozen in 500 μL plastic straws was obtained from Genex Cooperative, Inc. (Ithaca, NY, United States, prior to its closure in 2021.) and stored in liquid nitrogen. Before use, the straws were thawed in a 37°C water bath for 30 sec. Subsequently, the sample was centrifuged through two layers (40% and 80%) of Bovipure in Bovidilute solution (Spectrum Technologies, Inc., Healdsburg, CA, United States) at 300 × g for 10 min. The supernatant was removed, and the pellet of sperm was suspended in 3 mL TALP, then centrifuged at 300 × g for 3 min. Following supernatant removal, the sperm pellet was re-suspended in 300 μL TALP and placed in an incubator at 38.5°C under 5% CO_2_ in humidified air.

### Construction of microfluidic device

The design of the silicon master mold was adopted from our previous work^12^. The device contained a channel 4 cm long, 2.47 mm wide, and 60 03BCm, 120 μm or 250 μm deep. The structure was made by SU-8 negative resist with one layer of photolithography. The usage of SU-8, instead of etching, is crucial in this application in order to have clean, sharp edges. The master mold was treated with (1H,1H,2H,2H-perfluorooctyl) trichlorosilane (FOTS) to aid in easily releasing PDMS from the silicon master. Microfluidic devices were cast onto PDMS (10:1 PDMS base to curing agent) (SLYGARD 184 Silicone Elastomer kit, Dow Corning, Midland, MI, United States) on the silicon master. Subsequently, the PDMS mixture underwent 30 min of degassing to remove all air bubbles and was cured at 65°C for 1 hr. Sperm seeding and fluid input ports were created by punching holes in PDMS using biopsy punches (Sklar, West Chester, PA, United States). The PDMS components were then securely bonded to glass slides after oxygen plasma treatment (HARRICK PLASMA, PDC-32G, Ithaca, NY, United States) using high RF power for 60 sec. The channels were filled with viscoelastic fluid, which was equilibrated at 38.5°C under 5% CO_2_ in humidified air for at least 2 hr before experiments. For the experiments, the microfluidic devices were placed in an environmentally controlled chamber (operated by OKO-Touch), which was kept at 38.5°C and humidified. Sperm were seeded into one end of the microfluidic device to allow them to swim into the channel.

### Visualization of flagellum interaction with a solid surface

A Nikon Eclipse inverted phase contrast microscope, equipped with a Hamamatsu ORCA Flash 4.0 V3 camera, was used to capture images. The videos were recorded using NIS Element BR software, with each video lasting 1 min. A microfluidic device featuring a sharp L-shaped corner was filled with 1% MC. No external flow was induced within the device. The experimental setup is illustrated in Supplementary Fig. 3. Videos of sperm moving close to one of the upper corners were captured using a 20× objective and a frame rate of > 150 frames per sec (fps). Subsequently, we used ImageJ tracking software (open source, National Institutes of Health) to manually analyze the movement of both the head and the flagellum of sperm.

### Flow field measurement

Using high-speed video microscopy, video sequences capturing both sperm movement and the motion of the tracers (aggregates of polystyrene beads, 0.35 μm, carboxylated) suspended in the 1% MC within a microfluidic device of 120 μm were recorded with a 20× objective at a frame rate of 250 fps. The movement of the tracers was analyzed using ImageJ, viewing the beads highly magnified so that individual pixels were easily visible. The beads (or the aggregates of the beads) showed up either brighter or darker than the background, and both cases were tracked. A special feature is typically used to reliably identify the same pixel of the bead from frame to frame. No external flow was induced in the device to isolate and analyze the effects of sperm movement on the surrounding fluid. The sperm head positions and the tracer bead locations were tracked using the Manual Tracking plugin in ImageJ from around 100 different cells from separate video recordings of sperm. The results from the tracking were analyzed using MATLAB. To map the flow field, the area surrounding the sperm was segmented into bins based on the relative positions of the tracer particles, and their velocities in each bin were averaged. The overall flow field was then visualized by assembling these average velocities from all bins, providing a clear map of the fluid movement influenced by sperm (see Fig. 3).

### Velocity profile measurement

Suspensions of 0.35 μm carboxylated polystyrene in 1% methylcellulose dissolved in TALP and 1 μm polystyrene beads in TALP control medium were introduced into a device with a channel depth of about 60 μm at a constant flow rate of 1.5 μl/min using a syringe pump. Videos capturing the movement of each fluid were recorded at various Z-positions, using a 20× objective at a capture rate of 20 fps. The video sequences were then analyzed using ImageJ software, which facilitated direct tracking of the respective tracer beads. The software provided instantaneous velocities of each bead by calculating their displacement over time between frames. These velocities were then averaged across several beads in different regions of the channel at different depths to construct the velocity profile of each fluid.

## Data Availability

The data that support the findings of this study are available from the corresponding author upon reasonable request.
